# [^68^Ga]Ga-Schizokinen, a Potential Radiotracer for Selective Bacterial Infection Imaging

**DOI:** 10.1021/acsinfecdis.4c00067

**Published:** 2024-07-16

**Authors:** Asma Akter, George Firth, Afnan M. F. Darwesh, Margaret S. Cooper, Hataichanok Chuljerm, Agostino Cilibrizzi, Philip J. Blower, Robert C. Hider, Oliver Lyons, Silke Schelenz, Varun Mehra, Vincenzo Abbate

**Affiliations:** † Institute of Pharmaceutical Science, Faculty of Life Science and Medicine, King’s College London, London SE1 9NH, United Kingdom; ‡ School of Biomedical Engineering and Imaging Sciences, Faculty of Life Science and Medicine, King’s College London, London SE1 7EH, United Kingdom; § Department of Radiologic Sciences, Faculty of Applied Medical Sciences, King Abdulaziz University, Jeddah 21589, Saudi Arabia; ∥ School of Health Sciences Research, Research Institute for Health Sciences, Chiang Mai University, Chiang Mai 50200, Thailand; ⊥ Department of Surgery, University of Otago, Christchurch 8013, New Zealand; # Department of Microbiology, Kings College Hospital NHS Foundation Trust, London SE5 9RS, United Kingdom; ∇ Department of Hematology, King’s College Hospital NHS Foundation Trust, London SE5 9RS, United Kingdom

**Keywords:** bacterial infections, infection imaging, PET/CT, gallium-68-labeled siderophores, radiotracer, diagnostics

## Abstract

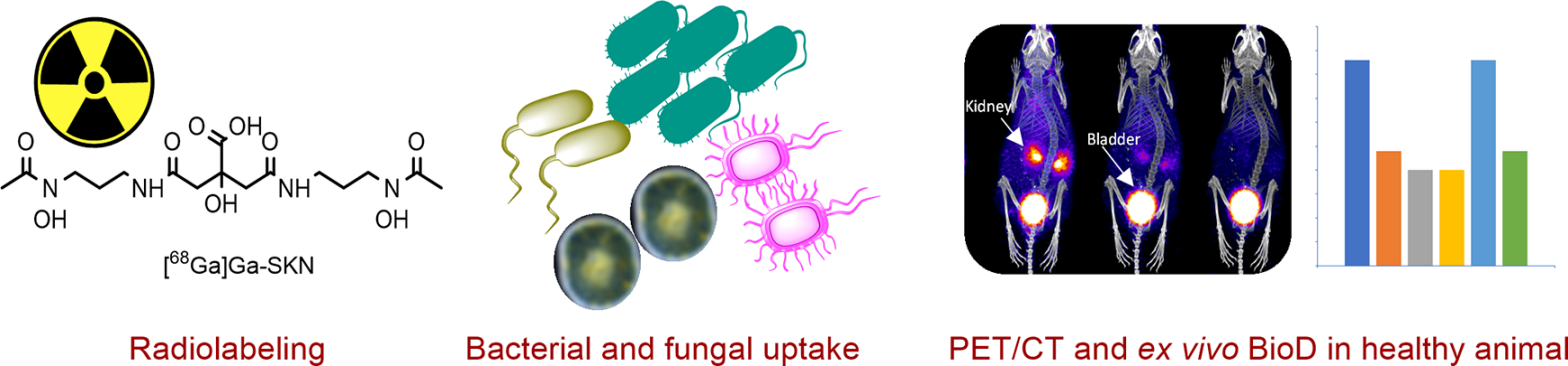

Gallium-68-labeled siderophores as radiotracers have gained interest for the development of *in situ* infection-specific imaging diagnostics. Here, we report radiolabeling, *in vitro* screening, and *in vivo* pharmacokinetics (PK) of gallium-68-labeled schizokinen ([^68^Ga]Ga-SKN) as a new potential radiotracer for imaging bacterial infections. We radiolabeled SKN with ≥95% radiochemical purity. Our *in vitro* studies demonstrated its hydrophilic characteristics, neutral pH stability, and short-term stability in human serum and toward transchelation. *In vitro* uptake of [^68^Ga]Ga-SKN by *Escherichia coli*, *Pseudomonas aeruginosa*, *Staphylococcus aureus*, and *S. epidermidis*, but no uptake by *Candida glabrata*, *C. albicans*, or *Aspergillus fumigatus*, demonstrated its specificity to bacterial species. Whole-body [^68^Ga]Ga-SKN positron emission tomography (PET) combined with computerized tomography (CT) in healthy mice showed rapid renal excretion with no or minimal organ uptake. The subsequent *ex vivo* biodistribution resembled this fast PK with rapid renal excretion with minimal blood retention and no major organ uptake and showed some dissociation of the tracer in the urine after 60 min postinjection. These findings warrant further evaluation of [^68^Ga]Ga-SKN as a bacteria-specific radiotracer for infection imaging.

The diagnosis and treatment of bacterial infections are challenging because of the difficulties in isolating and identifying pathogens early, determining pathogen sensitivity to drugs, selecting the most effective treatment options, and monitoring the success of treatment over an appropriate duration. Treatment outcomes worsen when pathogens become resistant to commonly prescribed antimicrobial agents.^1^ In 2019, bacterial pathogens caused an estimated 7.7 million deaths globally, 1.27 million of which were directly linked to antimicrobial resistance (AMR).^2^*Escherichia coli*, *Acinetobacter baumannii*, *Staphylococcus aureus*, *Streptococcus pneumoniae*, *Klebsiella pneumoniae*, and *Pseudomonas aeruginosa* were the leading causes of death. Although the use of rapid molecular and genome-based diagnostic techniques is increasing,^3^ infection-specific *in situ* imaging as a diagnostic tool remains an unmet clinical need. This approach is particularly relevant for the early detection of invasive infections in immunocompromised patients, for locating and detecting microbial infections in the body (for example, infection of implanted prosthetics^4−6^), and for monitoring treatment efficacy. Total-body positron emission tomography (PET) combined with computerized tomography (CT) or single-photon emission computed tomography (SPECT) in clinical practice uses nonspecific radiotracers such as [^18^F]fluorodeoxyglucose (FDG), [^67^Ga]Ga-citrate, and indium-111- and technichium-99m-labeled leukocytes.^7−9^ None of these radiotracers can directly image the infection in the body but rather capture secondary host responses against infections.^10^

Research has been ongoing to identify microbial targets for developing sensitive and specific radiotracers for imaging infections.^11−13^ Among them, sorbitol analogue 2-[^18^F]fluorodeoxysorbitol (2-[^18^F]FDS) targeting the sugar metabolic pathway,^14^ radiolabeled D-amino acids (DDAs) targeting cellular components,^15^ and gallium-68 (^68^Ga)-labeled siderophores targeting the iron transport pathway^4^ are the front runners for bacteria-specific radiotracer developments. However, both 2-[^18^F]FDS and DDAs have recently shown preclinical imaging of fungal infections,^14,16^ thus rendering them broad-spectrum infection imaging agents. Interestingly, siderophores, which transport the essential transition metal iron(III) (Fe^3+^) into microbial cells from the surrounding environment, can be designed as bacterial- and fungal-specific radiotracers. Typically, Fe^3+^ transport is mediated by the recognition of siderophores via specific cell-surface receptors and the internalization of siderophore-bound iron by the active transportation system of the membrane, Siderophore-Iron-Transporters (SITs). These receptors are generally specific but can often recognize xenosiderophores (produced by other microbes) under iron-limited/iron-depleted conditions.^17−19^ During infection, pathogens increase the production of these low molecular weight siderophores with high affinity for iron to obtain Fe^3+^ because host proteins such as transferrin, lactoferrin, and ferritin tightly regulate the availability of iron in the host environment.^20^ Siderophore-mediated iron transport in microbes has been discussed in detail elsewhere.^21^

^68^Ga is a PET radioisotope that chemically resembles Fe^3+^. This allows microbes to import siderophores radiolabeled with ^68^Ga into microbial cells instead of Fe^3+^-siderophores. Different ^68^Ga-siderophores have been investigated preclinically for imaging infection, and PET/CT imaging in animal infection models has proven the utility of ^68^Ga-siderophores as diagnostic tools.^22^ Furthermore, the utilization of xenosiderophores offers the opportunity to develop both broad-spectrum and narrow-spectrum radiotracers that could be useful in relevant clinical cases, such as polymicrobial infections and vascular graft infections due to fistulae with the enteric or respiratory tract.

To date, though no siderophore-based radiotracers have been developed clinically, the FDA approved the siderophore-conjugated β-lactam cefiderocol to treat Gram-negative bacterial infections.^23^ Furthermore, the natural hydroxamate siderophore desferrioxamine B (DFO-B; Figure 1A) is already an approved drug for iron overload treatment.

**Figure 1:**
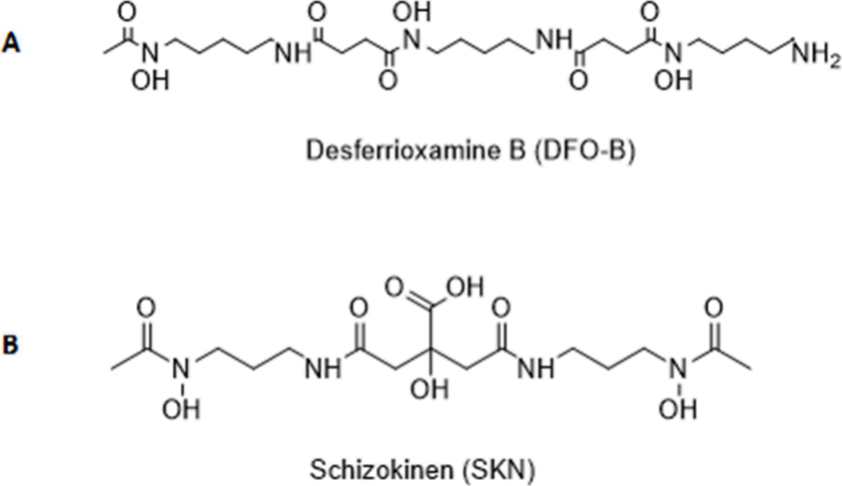
Chemical structure of desferrioxamine B and schizokinen.

Researchers have repurposed DFO-B by investigating its potential for imaging infections. It has turned out to be a broad-spectrum radiotracer for imaging bacterial and fungal infections.^24,25^ A phase I/IIa clinical trial using [^68^Ga]Ga-DFO-B for imaging the upper/lower respiratory tract or orthopedic bacterial infections is ongoing in Europe (EudraCT no. 2020-002868-31). Another small observational clinical study with [^68^Ga]Ga-DFO-B is being conducted to image vascular graft infections in the UK (ID: NCT05285072).

Schizokinen (SKN) is a citrate-based hydroxamate-siderophore produced by the Gram-positive bacterium *Bacillus megaterium* (Figure 1B). SKN is a low-molecular-weight (420 Da) negatively charged compound. SKN has been reported to possess a higher affinity for Fe^3+^ than DFO-B (pFe^3+^ value for SKN, 26.8 > DFO-B, 25.0).^26,27^*In vivo* studies of SKN revealed low bioavailability via the oral route, suggesting that SKN might not be a suitable candidate for iron overload treatment,^26^ similar to DFO-B. An earlier study characterized the coordination chemistry of gallium(III)-SKN and revealed the structural resemblance of Ga(III)-SKN to the iron analog Fe(III)-SKN.^28^ Therefore, owing to its comparable affinity for Fe^3+^, SKN deserves evaluation as an infection-specific PET tracer.

In this study, we established radiolabeling of SKN with ^68^Ga and evaluated the *in vitro* uptake of this complex by Gram-positive (*S. epidermidis, S. aureus*) and Gram-negative (*P. aeruginosa*, *E. coli*) bacteria and fungi (*Candida albicans*, *C. glabrata*, and *Aspergillus fumigatus*). Subsequently, we performed PET/CT imaging and determined the *in vivo* pharmacokinetics in a healthy animal model. As far as we are aware, our study is the first report on [^68^Ga]Ga-SKN in terms of radiolabeling and characterization, microbial uptake specificity, *in vivo* animal imaging by PET/CT, and *ex vivo* biodistribution (BioD).

We radiolabeled SKN with ^68^Ga, resulting in the [^68^Ga]Ga–SKN complex (Figure 2) with a radiochemical purity (RCP) of ≥95%, as confirmed using instant thin-layer chromatography (iTLC). The radiochemical yield (RCY) was 96.91 ± 2.76%, expressed as the percentage (%) of radioactivity in [^68^Ga]Ga-SKN (decay corrected) relative to the initial activity added in the reaction. Figure S1 shows radio-iTLC of [^68^Ga]Ga-SKN and [^68^Ga]Ga-acetate (negative control) captured using a Cyclone Plus Phosphor Imager (PerkinElmer). The retention factor (Rf) for [^68^Ga]Ga-SKN was 0.83, whereas [^68^Ga]Ga-acetate had an Rf value of 0–0.1 (determined by radio-iTLC). Figure S2A–F show radio-iTLC analyzed by iTLC scanner, RP radio-HPLC of [^68^Ga]Ga-SKN and [^68^Ga]Ga-acetate, and MS spectra for free SKN^26,29−31^ and cold-gallium SKN.^28^

**Figure 2:**
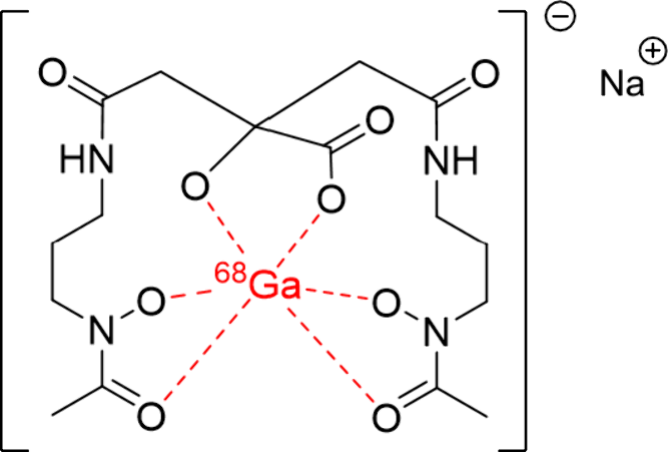
Proposed chemical structure of [^68^Ga]Ga-schizokinen in sodium acetate buffer.

The partition coefficient of [^68^Ga]Ga-SKN was expressed as log *D*_7.4_ and log *P* values.^32^ The log *D*_7.4_ and log *P* values for [^68^Ga]Ga-SKN were −2.43 ± 0.85 and −3.83 ± 0.15, respectively (Figure S3). For [^68^Ga]Ga-acetate the log *D*_7.4_ and log *P* values were −2.79 ± 0.15 and −1.83 ± 0.63, respectively. Partition coefficient studies of [^68^Ga]Ga-SKN demonstrated its hydrophilic nature, in line with other published ^68^Ga siderophores.^24,33−35^

*In vitro* stability in PBS was determined by incubating [^68^Ga]Ga-SKN and [^68^Ga]Ga-acetate (as a control) in PBS (1:1 v/v) and analyzing the samples by either radio-HPLC or radio-iTLC at 5 and 120 min. The tracer was stable in PBS (Figure S4A–C). With the human serum, the stability of [^68^Ga]Ga-SKN and [^68^Ga]Ga-acetate (as a control) was performed at short-time point (5 min incubation), which showed [^68^Ga]Ga-SKN was 100% stable in human serum after 5 min of incubation as determined by radio-HPLC (Figure S4D,E).

We also assessed the stability of [^68^Ga]Ga-SKN in terms of transchelation in the presence of an excess of the competitive chelator diethylenetriamine pentaacetate (DTPA; 6 mM). The results show that after 5 min, the radiotracer was ∼90% intact, and after 60 min, it was ∼50% intact (Figure S5). Our findings are similar to those reported for [^68^Ga]Ga-Ornibactin,^35^ which was found 80% intact after 30 min and 70% intact after 60 min, and [^68^Ga]Ga-DFO-B,^24^ which was 94% intact after 30 min and 85.3% intact after 60 min in the presence of 6 mM DTPA. However, the pH of the reaction for our DTPA study was not adjusted to a neutral value, resulting in an acidic environment. This is particularly important for [^68^Ga]Ga-SKN because SKN is pH-sensitive.^29,36^ Nevertheless, the transchelation study shows the complex is not inert to ligand exchange, although *in vivo*, the main competing ligand would be transferrin, not DTPA.^32^ Future work should investigate the incubation of the tracer at neutral pH in the presence of DTPA and at different time points in both DTPA and human serum. Furthermore, any breakdown products or metabolites of SKN will also need to be investigated, as well as their potential toxicity.

We evaluated the uptake of [^68^Ga]Ga-SKN by bacterial and fungal strains. We confirmed the stability of [^68^Ga]Ga-SKN in media by iTLC (alike Figure S1) before the uptake experiment. Previous studies have predicted the utilization of xenosiderophore SKN in *S. aureus* based on the presence of siderophore receptors.^37^ Accordingly, our study confirmed the high uptake of [^68^Ga]Ga-SKN by both *S. aureus* and *S. epidermidis*. The literature shows that *E. coli* K-12 is devoid of SKN-specific outer membrane receptors and that there is no *in vitro* utilization of SKN by this strain.^38^ However, high similarity in amino acid sequences between the Fe^3+^-aerobactin IutA transporter protein present in *E. coli* and the outer membrane transport protein Fe^3+^-SKN SchT in *Anabaena* sp. PCC 7120 has been previously reported.^39^ Thus, *E. coli* harboring aerobactin transporter proteins (*E. coli* may produce multiple siderophores, including enterobactin, aerobactin, yersiniabactin, and salmochelin)^40^ may also transport SKN. [^68^Ga]Ga-SKN uptake by the *E. coli* strain may be due to this intrastrain variability. The utilization of SKN in *P. aeruginosa* via the inner membrane protein FoxB, which is responsible for ferrichrome, ferrioxamine B, and SKN transport, has already been reported.^38^

*S. aureus* and *S. epidermidis* (Gram-positive) exhibited higher uptake (more than 100-fold in the case of *S. epidermidis*) than did *E. coli* and *P. aeruginosa* (Gram-negative) under iron-depleted conditions (Figure 3A), showing Gram-positive species selectivity of [^68^Ga]Ga-SKN. A previous study with [^68^Ga]Ga-DFO-B showed its higher uptake in *S. aureus* and *S. agalactica* compared to *P. aeruginosa*, and the level of uptake was variable among strains of the same bacterial species due to the STs upregulation affected by inherent genetic determinants or environmental cues.^24^ Here, we observed higher *in vitro* uptake of [^68^Ga]Ga-SKN in *S. epidermidis* than in *S. aureus.*

**Figure 3:**
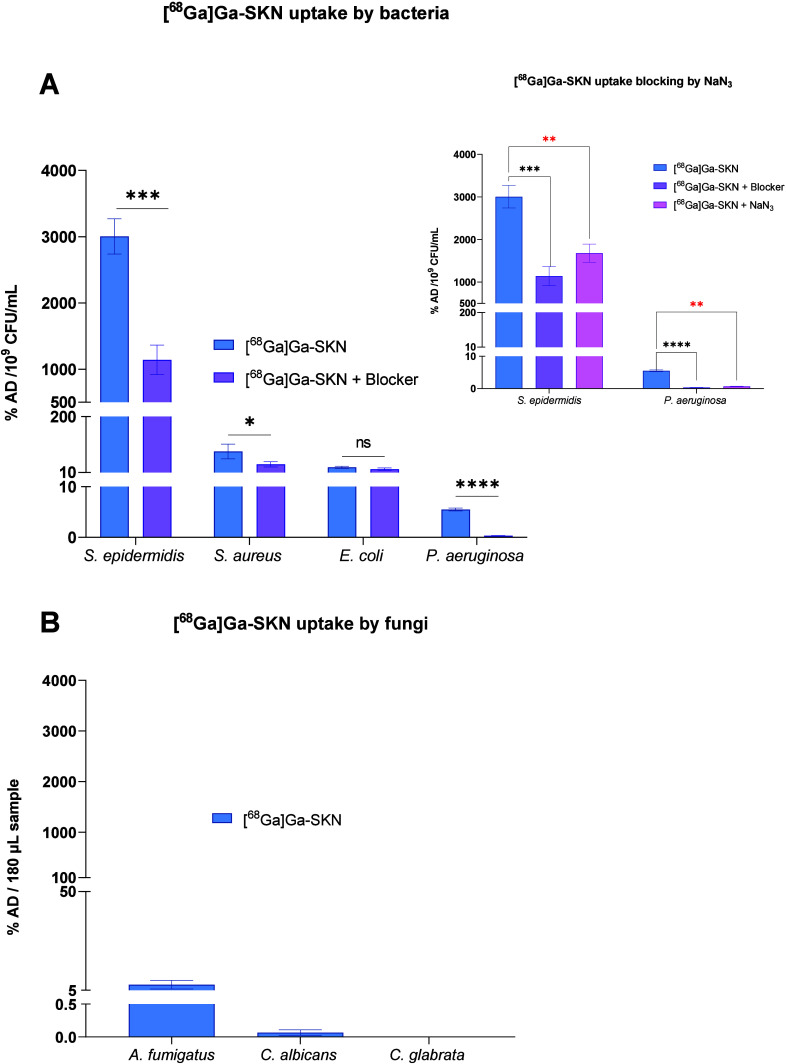
*In vitro* uptake of [^68^Ga]Ga-SKN by bacteria and fungi under iron-depleted conditions. (A) Uptake of [^68^Ga]Ga-SKN by Gram-negative and Gram-positive bacteria after 45 min of incubation (±Fe-ENT, 18 μM; ±Fe-PVD, 15 μM; ±Fe-SKN, 9 μM). Inset shows reduced uptake of [^68^Ga]Ga-SKN by *P. aeruginosa* and *S. epidermidis* in the presence of 30 mM NaN_3_. (B) Uptake of gallium-labeled siderophores by *C. albicans*, *C. glabrata*, and *A. fumigatus* after 45 min of incubation (as %AD). Each experiment was performed in triplicate (mean ± SD). *P* value < 0.05 was significant “*,” and “ns” means nonsignificant.

The uptake specificity of [^68^Ga]Ga-SKN in both Gram-negative and Gram-positive bacteria was determined by adding a blocking agent at excess concentrations, such as Fe-ENT, Fe-PVD, or Fe-SKN (Figure 3A). It was determined that these blocking agents were able to block up to 23% of [^68^Ga]Ga-SKN uptake in *E. coli* (Fe-ENT), 94% in *P. aeruginosa* (Fe-PVD), 30% in *S. aureus* (Fe-SKN), and 40% in *S. epidermidis* (Fe-SKN). It is speculated that this uptake could be blocked to a greater extent in *E. coli* and *S. aureus* with higher concentrations of the blocking agent in excess.

The cell viability after incubation with [^68^Ga]Ga-SKN was minimally affected compared to the control for all bacterial strains except for *S. epidermidis* (Figure S6). For *S. epidermidis*, a 1.7-fold reduction was observed. This inhibition is likely to occur because of intracellular iron deprivation (due to iron-limited condition), which could otherwise be used in metabolism.^41^ Additionally, literature shows that coagulase-negative staphylococci (such as *S. epidermidis*) are more susceptible to iron deprivation.^41,42^ Interestingly, viability was improved (about a 2-fold increase) in the presence of excess cold Fe-SKN in *S. epidermidis*. This may be due to the partial rescue of iron-deprived cells.

As [^68^Ga]Ga-SKN uptake was higher in *P. aeruginosa* and *S. epidermidis*, further investigations were performed to determine the role of the active transport system in this uptake. A previous study used NaN_3_ (2 mM) to block [^68^Ga]Ga–PVD-PAO1 uptake by *P. aeruginosa*.^33^ NaN_3_ is an inhibitor of ATP synthesis and affects microbial growth and metabolic activities.^43^*In vitro* uptake blockade of *S. epidermidis* and *P. aeruginosa* by the active transport blocker sodium azide (NaN_3_,) compared to the blocking agents is shown in the inset of Figure 3A. The results showed that 30 mM NaN_3_ blocked [^68^Ga]-Ga-SKN uptake by up to 87% in *P. aeruginosa* and up to 50% in *S. epidermidis*. The total viable count and raw radioactivity (cpm) are shown in Figure S7. Our findings indicate that [^68^Ga]Ga-SKN taken up by *P. aeruginosa* and *S. epidermidis* is an active transport-mediated uptake process by metabolically active cells.

*In vitro* uptake in 96-well plates by fungal strains, including *C. albicans*, *C. glabrata*, and *A. fumigatus*, was performed to assess the microbial specificity of [^68^Ga]Ga-SKN. Although yeast and mold cell types are different, the uptake results (%AD) presented here were obtained with the same volume of 180 μL of cells. For *C. glabrata* and *C. albicans*, 180 μL of sample had ∼1.8 × 10^6^ to 10^7^ CFU. The results showed almost no uptake by *C. glabrata*, *C. albicans*, and *A. fumigatus* (Figure 3B). Recent literature makes it evident that *A. fumigatus* is unable to utilize xenosiderophore, SKN, and, so far, the absence of SITs for SKN in *Candida* species.^44^ Hence, our results indicated that [^68^Ga]Ga-SKN uptake is specific to clinically relevant bacterial species, which could distinguish between clinically relevant bacterial and fungal infections.

Encouraged by having shown that Ga^3+^ is complexed by SKN, that the complex is stable at neutral pH, its short-term stability in the presence of excess DTPA and in human serum, that the complex is selectively taken up by certain microbial species, and the complex is expected to circulate *in vivo* before being renally excreted rapidly (based on the known behavior of other ^68^Ga-siderophore complexes),^24,25,32^ we further investigated *in vivo* pharmacokinetics of [^68^Ga]Ga-SKN as a potential bacterial-specific radiotracer. MicroPET/CT imaging of healthy BALB/c mice injected with ^68^Ga-labeled SKN revealed rapid clearance from the bloodstream, with major excretion by the renal system (Figure 4). This contrasts with the behavior of unchelated gallium, which shows prolonged circulation due to rapid binding to transferrin.^32^ Minimal retention was observed in the blood and other organs. Region of interest (ROI) data from PET imaging over 60 min are shown in Figure S8, which shows rapid circulation in the body and gradual accumulation of the radioactivity in the bladder. These data suggest that the biological half-life of [^68^Ga]Ga-SKN is ∼7 min, which is similar to other published ^68^Ga siderophores.^24,45^

**Figure 4:**
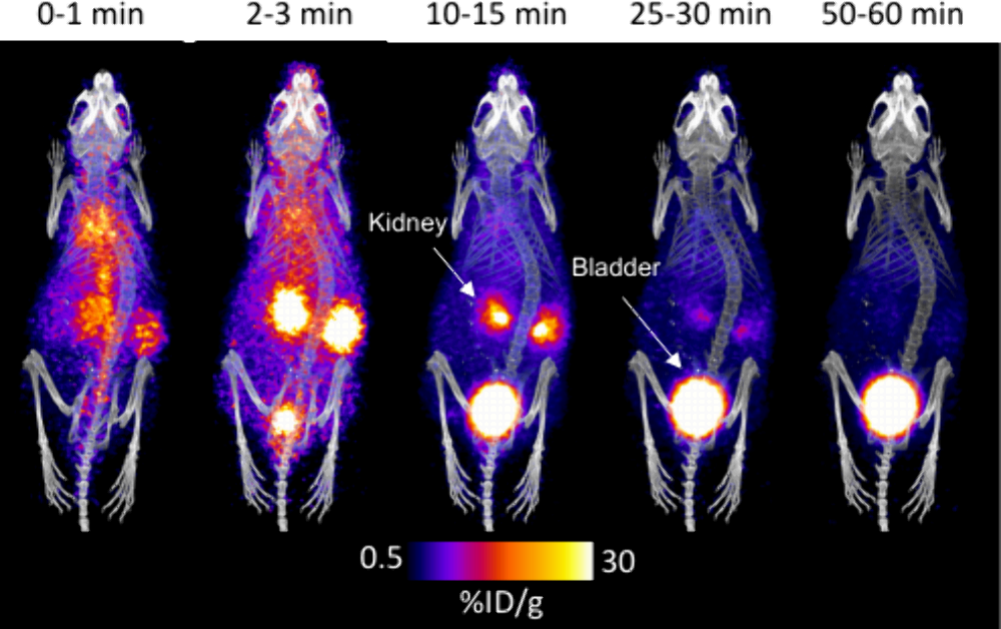
Dynamic PET/CT maximum intensity projection (MIP) images of healthy BALB/c mice (*n* = 4) 60 min after injection of [^68^Ga]Ga-SKN (2 MBq, intravenous injection via the tail). PET/CT images show the fast renal excretion of [^68^Ga]Ga-SKN.

The *ex vivo* BioD values were in accordance with the data obtained from PET/CT imaging 60 min postinjection (p.i.). Rapid bloodstream clearance of [^68^Ga]Ga-SKN was observed, with the renal system accounting for the majority of its excretion (Figure 5). There was minimal retention in the blood and other organs at 60 min p.i. The highest activity after 60 min was found in the kidneys (excluding bladder as gradual accumulation occurred there), at 5.37 ± 0.85% of the injected dose per gram of organ (ID/g). Similar findings were also highlighted by the iron–SKN complex *in vivo*, where iron-SKN was rapidly cleared from the bloodstream.^26^ Furthermore, this rapid PK is comparable with those of other reported ^68^Ga siderophores, which were later evaluated for imaging infections in small animal models.^24,33−35^

**Figure 5:**
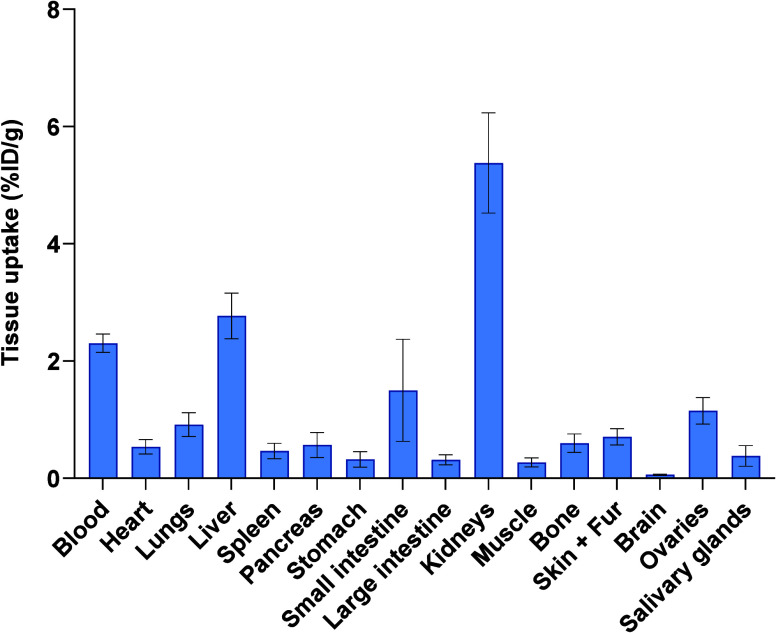
*Ex vivo* biodistribution of [^68^Ga]Ga-SKN in healthy BALB/c mice 60 min postinjection (p.i.). The mice were intravenously injected with 2 MBq of [^68^Ga]Ga-SKN via the tail vein (*n* = 4). Error bars show the standard deviation: % ID/g, percentage of injected dose per gram of organ.

Radio-HPLC and radio-iTLC analysis of urine showed that [^68^Ga]Ga-SKN was excreted largely in the urine sample collected 60 min p.i., and there is some presence of unchelated gallium-68, which indicates it may have been dissociated from the [^68^Ga]Ga-SKN over the time (Figure S9). This result is similar to [^68^Ga]Ga-DFO-B,^32^ which has been shown to be a broad-spectrum infection-specific radiotracer to detect Gram-positive, Gram-negative, and fungal pathogens in relevant lung and muscle animal infection models. Therefore, we envisage that [^68^Ga]Ga-SKN would be able to detect pathogens in *in vivo* infection models.

Direct imaging of live bacterial or fungal pathogens can reveal local infections in the body without the cumbersome and invasive direct sampling used in traditional slow-throughput infection diagnostics. *In situ* identification of causative pathogens will aid in distinguishing between bacterial and fungal pathogens, reduce the turnaround time to prescribe appropriate antimicrobial treatment, and improve patient outcomes. Moreover, monitoring the success of antimicrobial treatments will aid in addressing the AMR crisis.

We studied [^68^Ga]Ga-SKN radiosynthesis, its PBS stability, transchelation, short-term stability in human serum, and *in vitro* uptake in bacterial and fungal cells, which demonstrated bacterial specificity. Further PET/CT imaging and *ex vivo* BioD in healthy animals demonstrated the rapid clearance of this radiotracer from the blood by the renal system with very little retention in the major organs and blood circulation. *In vivo* healthy animal urine analysis shows ∼22% stability of [^68^Ga]Ga-SKN after 60 min p.i. collection, where free or unchelated ^68^Ga-acetate is present, similar to other ^68^Ga-siderophore studies. The limitations of the study are the short-term *in vitro* human serum stability test and ∼50% stability after 60 min in the presence of transchelator DTPA. However, based on published ^68^Ga siderophores’ *in vitro* and *in vivo* behaviors and their ability to detect pathogens, this brief period should be enough to reach the site of infection and accumulate inside microbial cells. Therefore, future work will include further investigation of its *in vitro* stability in human serum at different time points, a pH-adjusted transchelation assay, *in vivo* evaluation of [^68^Ga]Ga-SKN to detect bacterial infections (and to distinguish between bacterial and fungal infections) in clinically relevant infection models in small animals, and whether further chemical modifications are needed to alter its PK.

## Materials and Methods

All chemicals, culture media, and analytical grade reagents were purchased from Sigma–Aldrich (UK; unless otherwise stated). Siderophores, including desferrioxamine B (DFO-B), pyoverdine (PVD), and enterobactin (ENT), were purchased from Sigma–Aldrich (UK). Schizokinen (SKN) was isolated and purified from *Bacillus megaterium*(26) and/or obtained via chemical synthesis.^27^

Microbial strains were purchased commercially, except *for Candida albicans* (ATCC 90028), *Candida glabrata* (ATCC 90030), and *Aspergillus fumigatus* (ATCC 46640), which were kindly provided by Dr. Silke Schelenz, KCH Clinical Lead Infection Sciences, King’s College Hospital, London, UK. All of the strains and their growth media and temperatures, assay media, and blocking agents are listed in Table S1. Methods for the microbial uptake assay are described in the Supplementary Methods.

## Analytical Methods

Instant Thin Layer Chromatography (iTLC) was performed with a glass microfiber paper strip that is impregnated with silica gel (SG; Agilent Technologies, UK; mobile phase: 10% ammonium acetate, 30:70% water/methanol, or 50% water/methanol). Post-iTLC, strips were subjected to scanning using a Raytest Rita-Star TLC scanner with a positron (β+) detector (LabLogic, UK) and analyzed using Laura software (LabLogic, UK) or a Cyclone Plus Phosphor Imager (PerkinElmer). An Agilent Eclipse XDB C18 (5 μm 4.6 × 150 mm in diameter) reversed phase (RP) column was used for high-performance liquid chromatography (HPLC) along with UV detection at 220 nm (Gina Star TM software version 5.8). Further analysis was performed using Laura software (LabLogic, UK). Details of the composition of the mobile phase and gradients for RP-HPLC are mentioned in the Supplementary Methods. Radioactivity was measured with a gamma counter (LKB Wallac 1282 CompuGamma Gamma Counter or a PerkinElmer 3470 Wizard2 Gamma Counter).

## Radiosynthesis of [^68^Ga]Ga-SKN

A total of 5 mL of 0.1 M ultrapure HCl was introduced to a ^68^Ge/^68^Ga generator (Eckert and Ziegler Eurotope GmbH, Berlin, Germany) to elute ^68^GaCl_3_ in five tubes following the fractionated elution approach.

Radiolabeling with ^68^Ga was first optimized with variable amounts (μg) of SKN under different reaction conditions (pH, temperature, etc.). Briefly, 10–50 μg of SKN (1–4 μg/μL in water) was mixed with 60 μL of 3.6 M sodium acetate and 100–200 μL of eluted ^68^GaCl_3_ (10–120 MBq) and incubated at room temperature for approximately 10–15 min. The pH of the reaction mixtures was then adjusted to 6–7 with the addition of extra sodium acetate. The radiochemical purity (RCP) of [^68^Ga]Ga-SKN was confirmed using instant thin-layer chromatography (iTLC), as described in the Analytical Methods section.

## Characterization of [^68^Ga]Ga-SKN

The distribution coefficient was performed following the shake flask method.^32^ The PBS, human serum, and DTPA stability of [^68^Ga]Ga-SKN were determined via RP-HPLC. All of these protocols are described in the Supplementary Methods.

## Animal Experimental Design

An *in vivo* animal study was performed following the Animals (Scientific Procedures) Act, 1986. Appropriate project and personal licenses were approved by the UK Home Office. King’s College London Animal Welfare and Ethical Body approved these animal experiment protocols. Healthy female Balb/b mice (10–11 weeks, 10–12 g) were purchased from Charles River UK Ltd. The experiment was designed with four mice to evaluate the *in vivo* PK and *ex vivo* BioD of the radiotracer. Mice were intravenously injected with [^68^Ga]Ga-SKN (110–120 μL in PBS, 4–6 MBq, and 5.5–6.0 μg of SKN per animal) and filter sterilized with Millex-LG 0.20 μm prior to injection.

## PET/CT Imaging

The NanoScan PET/CT (Mediso Medical Imaging Systems) was employed for dynamic PET scanning and CT scanning.^32^ All of the images were rebuilt by Tera-Tomo (Monte Carlo-based full 3D iterative software). All of these rebuilt images were analyzed by VivoQuant 1.21 software (inviCRO), and quantification for regions of interest (ROIs) was performed for specific mouse tissues. Mice were anesthetized with isoflurane (1.0–1.5 L/min oxygen flow rate and 2–2.5% isoflurane) prior to cannulation of the tail vein, after which a CT scan was performed. Later, the radiotracer was injected as stated above, and a dynamic PET scan was performed for 1 h.

## *Ex Vivo* BioD

After PET/CT scan, animals were humanely killed by neck dislocation under anesthesia. Animal organs and tissues were harvested and weighed, and radioactivity was quantified using a gamma counter (for *ex vivo* BioD). These results were represented as “the percentage of the calculated injected dose per gram of relevant organ (% ID/g).” A mouse urine sample was collected from the bladder to evaluate the stability of [^68^Ga]Ga-SKN in the urine. The sample (100 μL) was analyzed using analytical RP-HPLC.

## Statistical Data Analysis

All data were processed using Microsoft Office Excel 2019. All of the figures presented with error bars (standard deviation) were created using GraphPad Prism 10. An “unpaired *t* test” for statistical significance was also performed in GraphPad Prism 10 to calculate *P* values (a *P* value equal to or less than 0.05 indicating statistical significance).

## Supporting Information Available

Supplementary Methods; microbial strains, growth and media (Table S1); [^68^Ga]Ga-SKN radiolabeling (Figures S1, S2A–D); MS spectra of free SKN and cold-gallium complex (Figure S2E,F); hydrophilicity determination (Figure S3); PBS and serum stability (Figure S4A–E); transchelation (Figure S5); viable cell count (Figure S6); effect of sodium azide (Figure S7); ROI quantification (Figure S8); urine stability (Figure S9) (PDF)
