# Supplementary material for: [68Ga]Ga-Schizokinen, a Potential Radiotracer for Selective Bacterial Infection Imaging
Source: ACS Infect Dis. 2024 Jul 16;10(8):2615–22. doi: 10.1021/acsinfecdis.4c00067 (PMC11320569; doi:10.1021/acsinfecdis.4c00067)
Supplement: Supplementary file 1 — id4c00067_si_001.pdf [file id4c00067_si_001.pdf]

Supplementary Information (SI)

Title: [^{68}Ga]Ga-Schizokinen, a potential radiotracer for selective bacterial infection imaging

Short title: [^{68}Ga]Ga-Schizokinen as a bacteria-specific PET tracer

Authors and affiliation:

Asma Akter[1*], George Firth[2], Afnan M. F. Darwesh[2&3], Margaret S. Cooper[2], Hataichanok Chuljerm[1&4], Agostino Cilibrizzi[1], Philip J. Blower[2], Robert C. Hider[1], Oliver Lyons[5], Silke Schelenz[6], Varun Mehra[7], Vincenzo Abbate[1*]

[1]Institute of Pharmaceutical Science, Faculty of Life Science & Medicine, King's College London, London, SE1 9NH, UK

[2]School of Biomedical Engineering & Imaging Sciences, Faculty of Life Science & Medicine, King's College London, London, SE1 7EH, UK

[3]Department of Radiologic Sciences, Faculty of Applied Medical Sciences, King Abdulaziz University, Jeddah, 21589, Saudi Arabia

[4]School of Health Sciences Research, Research Institute for Health Sciences, Chiang Mai University, Chiang Mai, 50200, Thailand

[5]Department of Surgery, University of Otago, Christchurch, 8013, New Zealand

[6]Department of Microbiology, Kings College Hospital NHS Foundation Trust, London, SE5 9RS, UK

[7]Department of Hematology, King's College Hospital NHS Foundation Trust, London, SE5 9RS, UK

Corresponding authors: asma.akter@kcl.ac.uk, vincenzo.abbate@kcl.ac.uk

Supplementary information:

Pages: 13

Tables: 1

Figures: 9

Supplementary Methods

Distribution coefficient

The hydrophilicity was determined using the shake flask method.[1] 0.3–0.5 MBq of the [^{68}Ga]Ga-SKN and [^{68}Ga]Ga-acetate (control) mixtures were added to a preequilibrated mixture of equal volumes of octanol and PBS (pH 7.4) (500 µL/500 µL) and octanol and water (500 µL/500 µL) in triplicate. The samples were vortexed for 2 min and then left to obtain good separation between the two layers. Two hundred microliters were taken from each layer, and their activity was measured separately with a gamma counter. The distribution coefficients logD and logP were calculated using the following equations:

$LogD_{pH\ 7.4}$=log ([radioactivity in octanol]/[radioactivity in PBS])

LogP=log ([radioactivity in octanol]/[radioactivity in water])

Stability in PBS and Serum

The [^{68}Ga]Ga-SKN and [^{68}Ga]Ga-acetate (control) mixtures were diluted (1:1 v/v) with PBS and incubated for 5 and 120 min at 37°C. Aliquots from the sample were analyzed directly by either reverse phase (RP) radio-HPLC or radio-iTLC.

The [^{68}Ga]Ga-SKN and [^{68}Ga]Ga-acetate (control) mixtures were diluted (1:1 v/v) with human serum and incubated for 5 min at 37°C. Ice-cold acetonitrile was added (1:1 v/v), and the samples were vortexed for 1 minute followed by centrifugation at 13,000 rpm for 5 minutes. Aliquots from the supernatant were used for RP-HPLC analyses after being diluted five times with water.

The RP-HPLC mobile phases used were water (A) and acetonitrile (B), (alternatively A: water, B: acetonitrile each containing 0.1% trifluoracetic acid (TFA)). Gradient: (0–2 min: 0% B, 2–24 min: 60% B, 24–26 min: 60% B, 26–28 min: 0% B).

Transchelation with diethylenetriamine pentaacetate (DTPA)

[^{68}Ga]Ga-SKN was mixed with 6 mM DTPA (1:1) and incubated for 5 min and 60 min at 37°C. Aliquots of the supernatant were subjected to RP-HPLC analysis (the HPLC conditions as described above). The pH of assay was not adjusted to neutral pH.

Microbial cell preparation

Bacterial cells were prepared following a previously optimized protocol by Akter et al. (in submission). Briefly, cultures were grown at 37°C with shaking at 200 rpm for 16–21 h (considered iron-depleted cells). A further 4h of incubation in fresh media were followed if required. Iron-replete cells with the addition of 10 µM $FeSO_4.7H_2O/FeCl_3$ were prepared in parallel. After washing with PBS and pelleting

down, cells were dissolved in pre-warmed (37°C) assay medium. One of our objectives was to develop radiotracers for vascular graft infections (VGEI). Clinical data suggests that the bacterial load is relatively low (10^2 CFU/mL), recovered from the infected grafts or stents[2]. We initially performed in vitro uptake studies in *E. coli* and *P. aeruginosa* with 10^9 CFU/mL. Then, we moved to S. aureus and coagulase-negative *S. epidermidis*. Since SKN is produced by the Gram-positive bacteria *Bacillus megaterium*, we expected its better uptake in other Gram-positive bacteria such as *S. aureus* and *S. epidermidis*. *S. epidermidis* being a critical pathogen for VGEI, we wanted to investigate the uptake of [^{68}Ga]-Ga-SKN with a low bacterial concentration (10^4 CFU/mL). *A. fumigatus* conidia (1 × 10^6 per mL) in 25 mL of assay media were inoculated and grown for 18–20 hours at 37°C with 180–200 rpm of shaking[3]. These cells are known as iron-depleted *A. fumigatus* cells. Parallel iron-replete cells were prepared by adding 30µM $FeSO_4$. $7H_2O$ /$FeCl_3$ to the assay medium.

Microbial uptake assay

In vitro uptake in bacterial cells was performed following a previously optimized protocol by Akter et al. (in submission). 20–50 µL (0.2–0.6 MBq) of [^{68}Ga]Ga-SKN was added to the iron-depleted and/or iron-replete cells and incubated for 45 min at 37°C with shaking at 180 rpm. Parallel control samples without the radiotracer were incubated in the same manner. After incubation, 1 mL sample from each tube was centrifuged at 6,000 ×g for 5 min, and pellets were washed with ice-cold PBS (3×). The activity in the pellets was measured using a gamma counter. The uptake results are expressed as a percentage of the added dose in 10^9 CFU per 1 mL culture (%AD/10^9 CFU).

For blocking or competition assays, bacterial cells were pre-incubated for 10–20 min at 37°C with Fe-ENT, Fe-PVD, and Fe-SKN (9–20 µM) or 30 mM sodium azide (NaN_3) prior to the addition of [^{68}Ga]Ga-SKN. Uptake assays were performed as described above.

Fungal uptake was performed in a 96-well filter plate (Millipore, Merck, UK).[2] 180 µL of iron-depleted cells was added to a prewetted 96-well filter plate and incubated with 20 µL of [^{68}Ga]Ga-SKN at 37°C with control. After 45 min of incubation, the plates were filtered and washed with ice-cold Tris (hydroxymethyl)aminomethane (Tris) buffer (3×). The dried filters were separated from the plate, and the radioactivity was measured by a gamma counter. The uptake results are expressed as a percentage of the added dose in 180 µL of culture (%AD 180 µL sample).

Total viability count

All the bacterial cell samples, including the control, were diluted in PBS to determine the viability of the cells after the uptake assays. 10-100 µL of diluted samples (10^{-6}, 10^{-5}, 10^{-4}, 10^{-3}, 10^{-2}) were either spotted or spread onto appropriate agar media and incubated at 37°C overnight in triplicate. Total cell

viability was expressed as colony-forming units (CFU)/mL. No viability assays were performed on the fungal strains.

Supplementary Tables

Table S1. Microbial strains, growth and assay media were used in this study.

Bacteria	Growth media	Growth temperature	Assay media	Blocking agent#
Escherichia coli NCIMB 10218	Luria–Bertani (Lenox) agar/broth	37℃	Minimal Medium 9 (MM9)*[4]	Fe-ENT (ENT is native)
P. aeruginosa NCTC 10662	Trypticase Soy agar/broth	37℃	Succinate Medium (SM)[5]	Fe-PVD (PVD is native)
Staphylococcus aureus NCTC 6571	Trypticase Soy agar/broth	37℃	Yeast Extract-Peptone-Glucose (YPG) Medium/ RPMI + 1 % (w/v) casamino acid[6,7]	Fe-SKN
Staphylococcus epidermidis NCIMB 8853	Trypticase Soy agar/broth	37℃	YPG Medium**[6]	Fe-SKN
Candida albicans ATCC 90028	Sabours Dextrose (SD), YPD Agar/broth	37℃	Glucose minimal medium (GMM)[8]	-
Candida glabrata ATCC 90030	SD, YPD Agar/broth	37℃	YPG Medium**/GMM[6,8]	-
Aspergillus fumigatus ATCC 46640	Malt extract agar (MEA) or SDA medium	25-28℃	Aspergillus minimal medium (AMM)*[3]	-

*Iron-replete conditions were obtained with the addition of 10 µM $FeSO_4.7H_2O/FeCl_3$ for bacteria and 30 µM for fungi.
**Iron-depleted conditions were obtained with the addition of 200 µM 2,2'-dipyridyne (DP).

#Blocking agents Fe-ENT, Fe-PVD, and Fe-SKN were prepared using $Fe_2(SO_4)_3.5H_2O$ or $FeCl_3.6H_2O$ with the desired siderophores at an equimolar ratio, unless stated otherwise. The complex was incubated at room temperature for 15 min, and the pH was adjusted to 6.5–7.0 with 10M sodium hydroxide (NaOH).

Supplementary Figures

Radiolabeling of [^{68}Ga]Ga-SKN

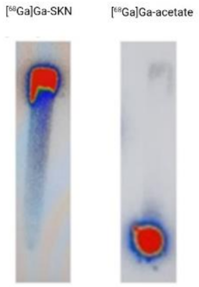

Figure S1. After radiolabeling of [^{68}Ga]Ga-SKN, instant thin-layer chromatography on silica gel impregnated glass fibers (iTLC-SG) (mobile phase 50:50 ammonium: methanol buffer) performed and images were captured by Phosphor imager (autoradiography).

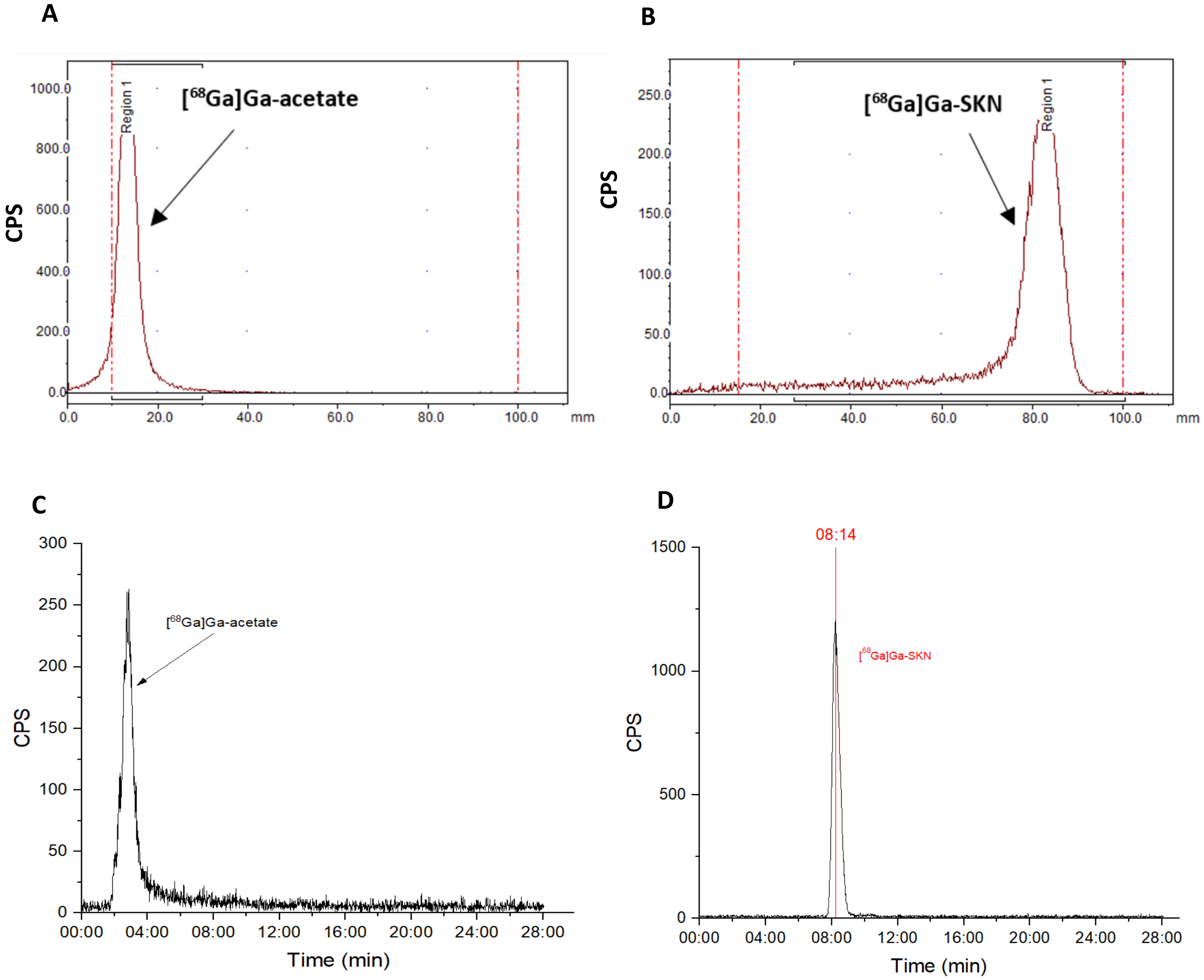

E

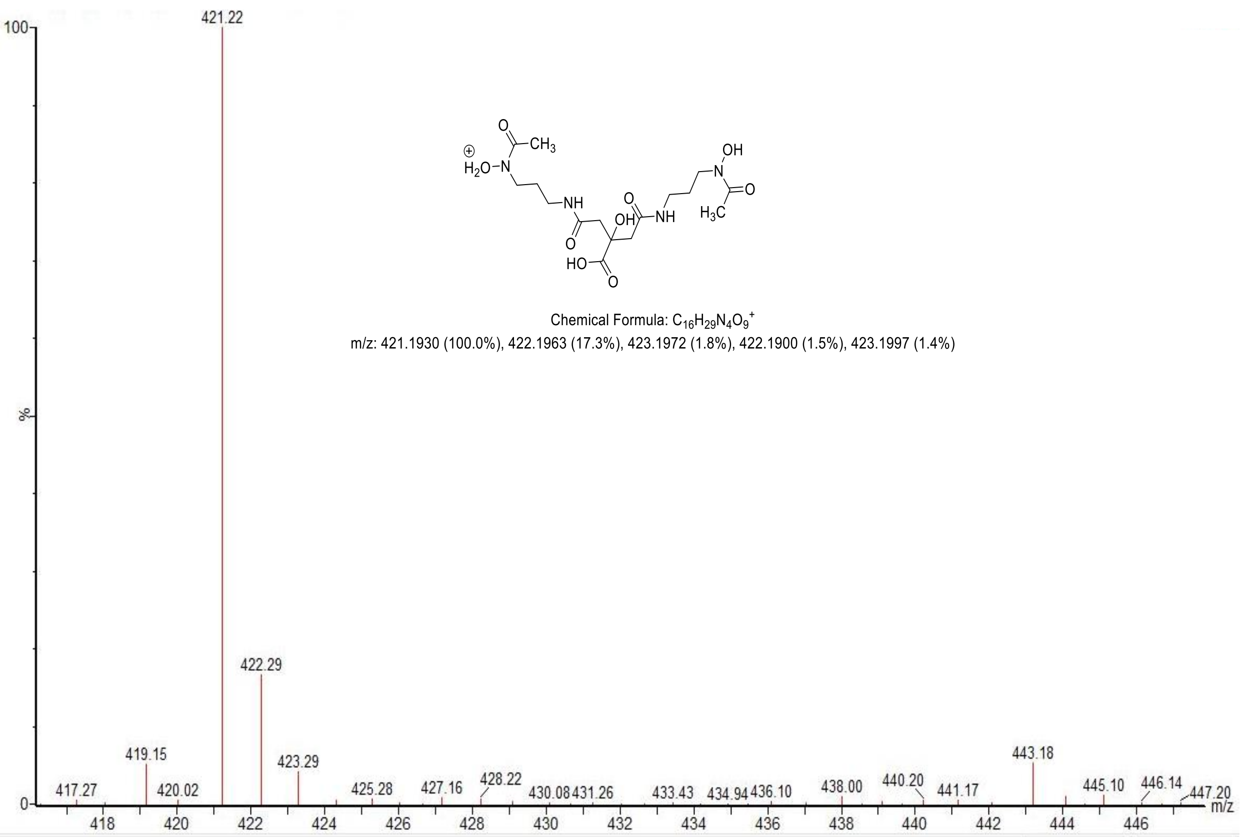

F

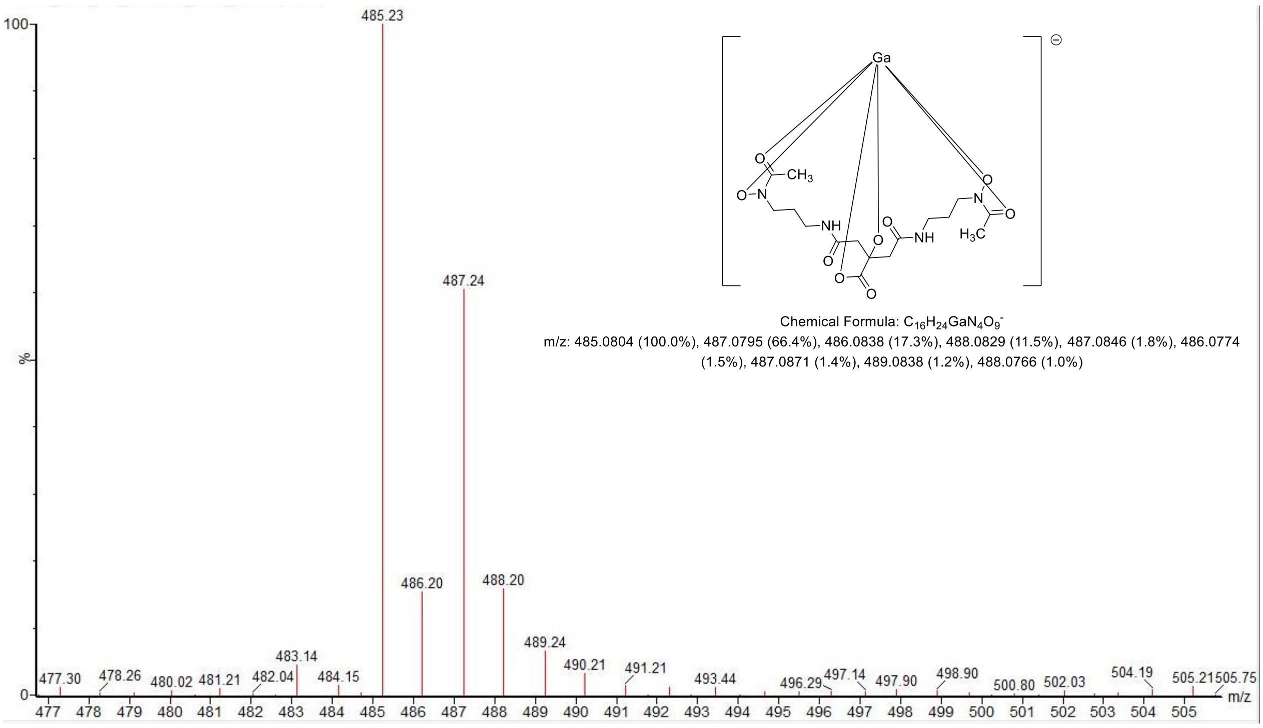

Figure S2. [^{68}Ga]Ga-SKN radiosynthesis analysis by radio-iTLC-SG scanner and RP radio-HPLC, and MS spectra for free SKN and cold-gallium SKN. (**A**) [^{68}Ga]Ga-acetate in iTLC scanner (**B**) [^{68}Ga]Ga-SKN in iTLC scanner,(**C**) [^{68}Ga]Ga-acetate in RP radio-HPLC, (**D**) [^{68}Ga]Ga-SKN in RP radio-HPLC,.[9](**E**) MS spectrum for free SKN (ESI+), and (**F**) MS spectrum for cold gallium-SKN (ESI-). CPS: counts per second.

LogP and LogD$_{7.4}$ value

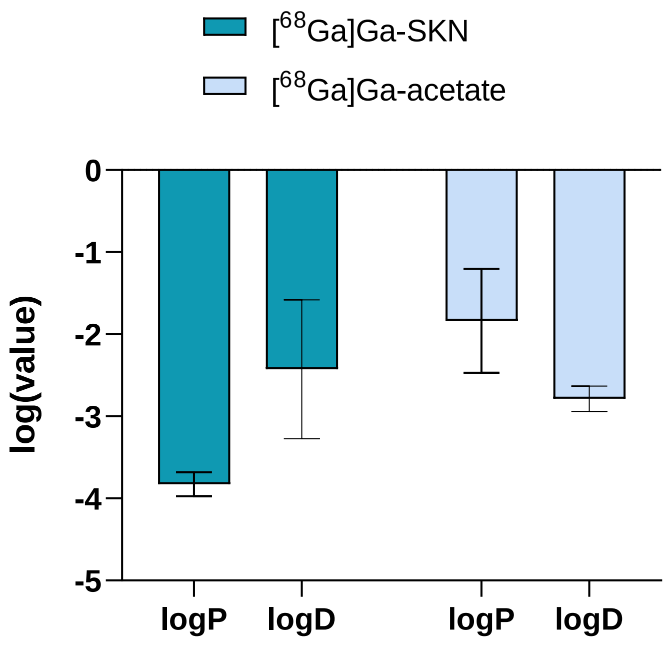

Figure S3. Hydrophilicity determination by logP and logD$_{7.4}$ value for [^{68}Ga]Ga-SKN and [^{68}Ga]Ga-acetate. Each experiment was performed in triplicate (mean ± SD).

PBS and Serum Stability of [^{68}Ga]Ga-SKN

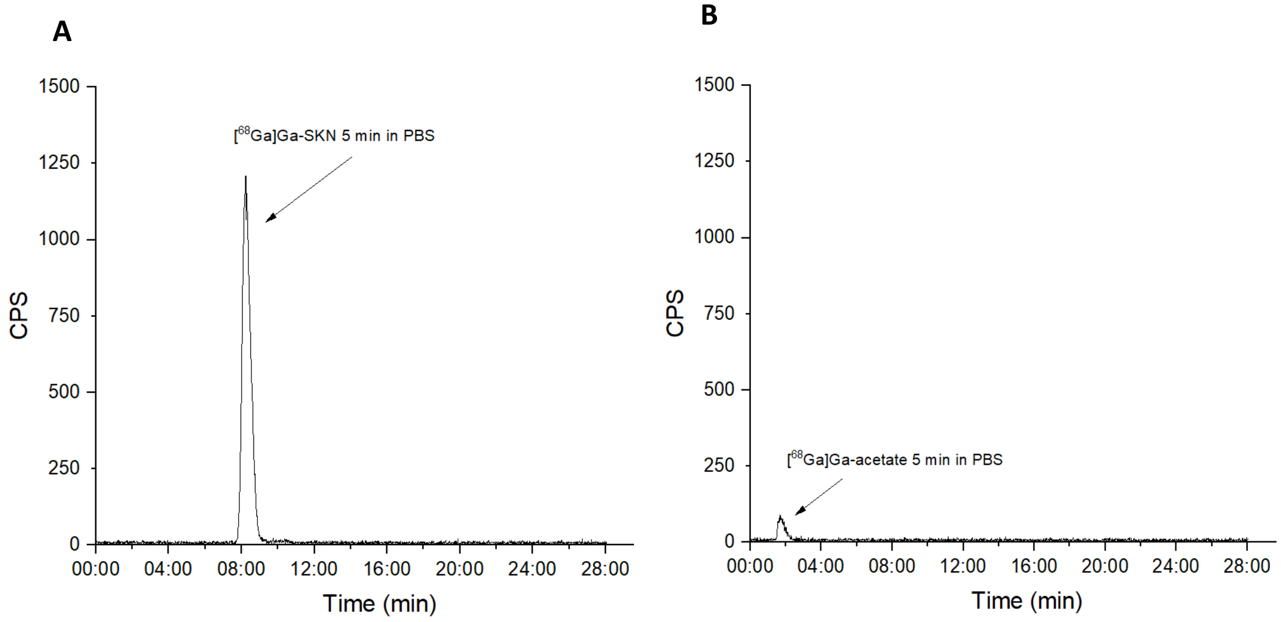

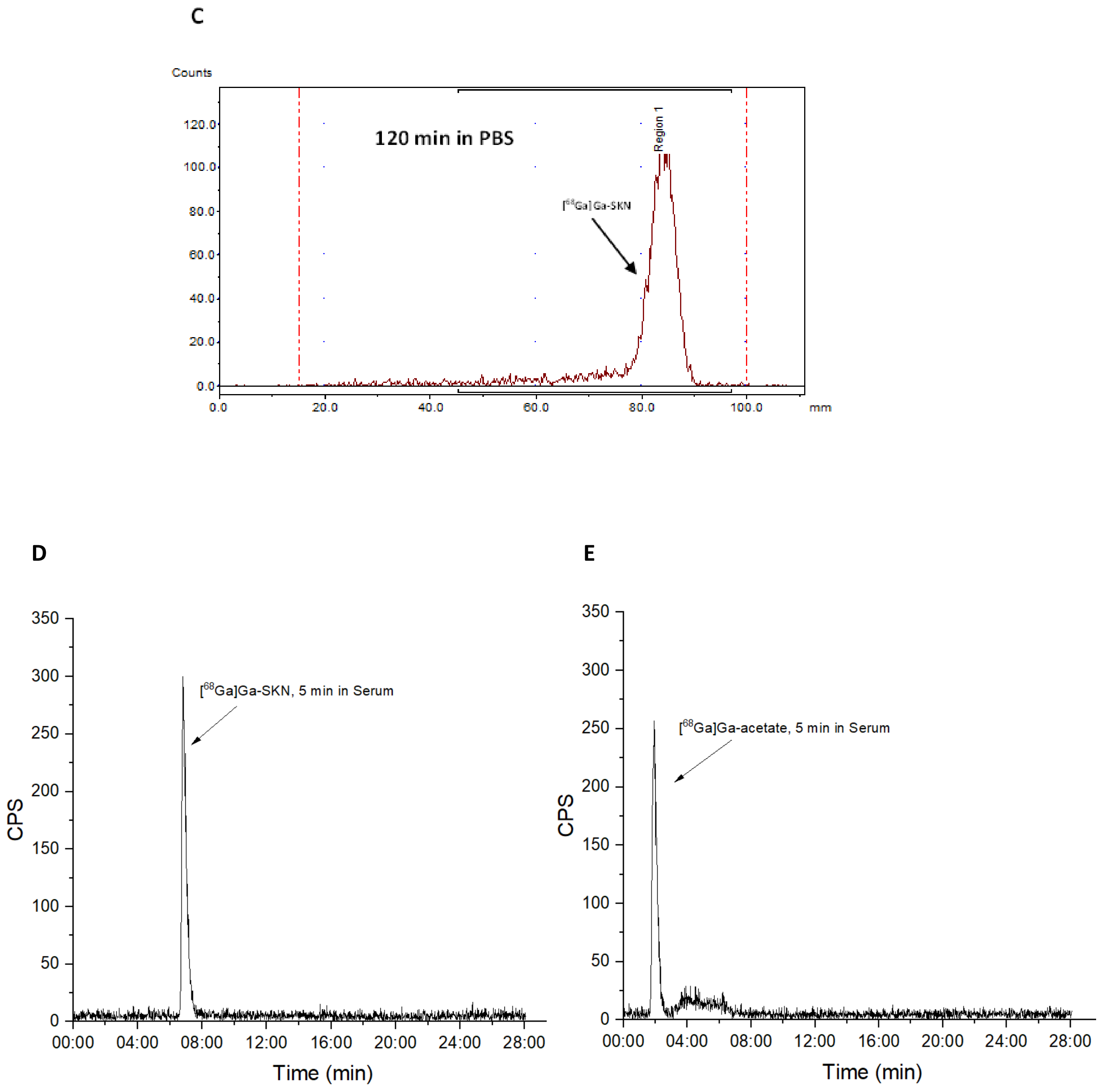

Figure S4. Stability of [^{68}Ga]Ga-SKN in PBS after 5 min of incubation by RP-HPLC (**A**), [^{68}Ga]Ga-acetate in PBS after 5 min of incubation by RP-HPLC (**B**), [^{68}Ga]Ga-SKN in PBS after 120 min of incubation by radio-iTLC-SG (**C**), [^{68}Ga]Ga-SKN in human serum after 5 min of incubation by RP-HPLC (**D**), [^{68}Ga]Ga-acetate in human serum after 5 min of incubation by RP-HPLC (**E**).CPS: counts per second.

Transchelation with DTPA

A

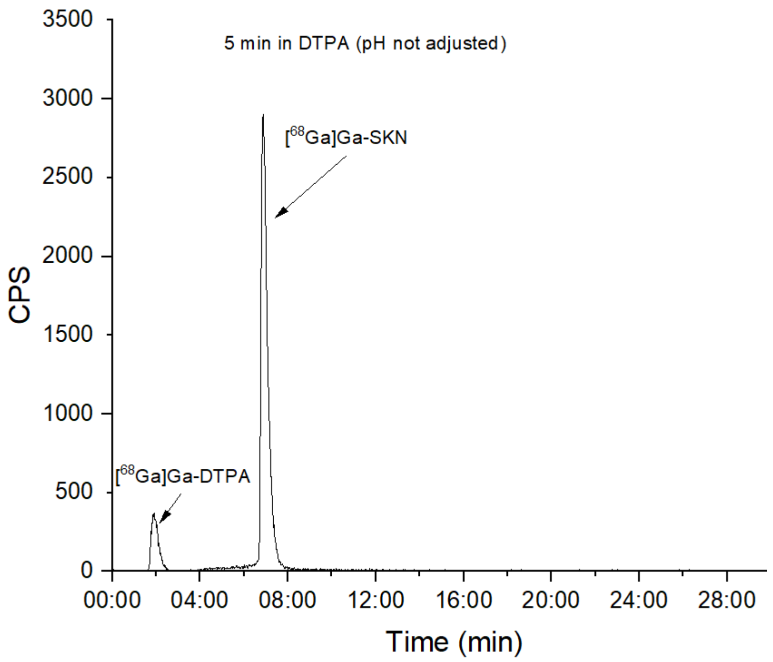

B

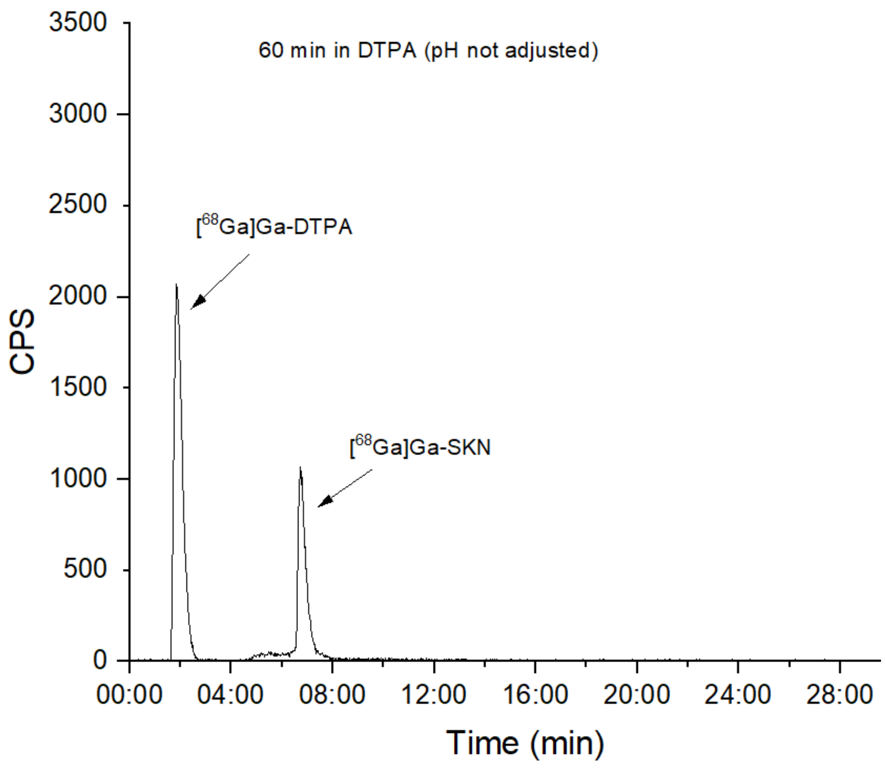

Figure S5. Stability of [^{68}Ga]Ga-SKN in in the presence of 6 mM DTPA (transchelation) (A) after 5 min, and (B) after 60 min of incubation was determined by RP-HPLC.

Viability count

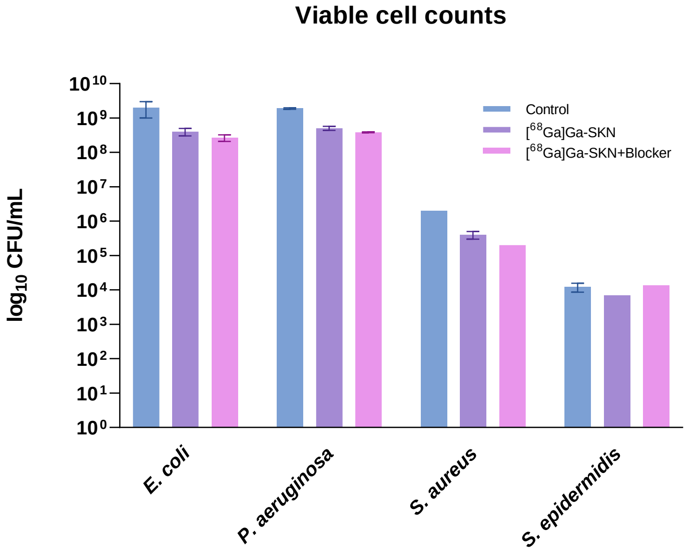

Figure S6. Total viability of bacterial cells after incubating with [^{68}Ga]Ga-SKN under iron-depleted conditions. Each experiment was performed in triplicate (mean ± SD). [^{68}Ga]Ga-SKN shows minimal effect on the viability of all the strains.

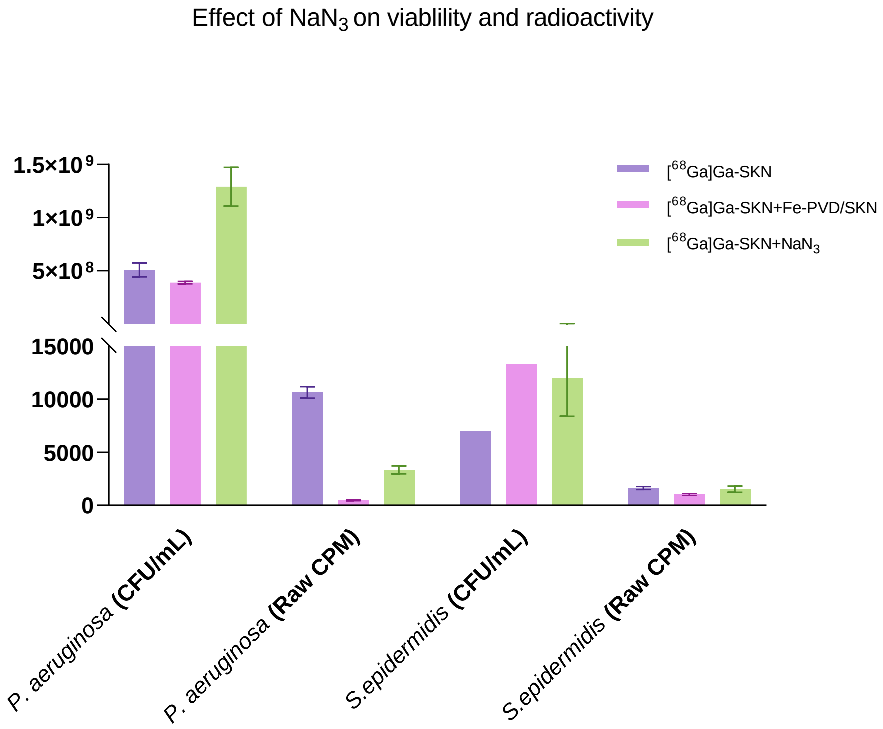

Figure S7. Effect of NaN_3 (30 mM) on the viability and uptake of radioactivity under iron-depleted conditions. Each experiment was performed in triplicate (mean ± SD). Results show no reduction in cell viability after NaN_3 treatment. CPM: counts per minute.

ROI

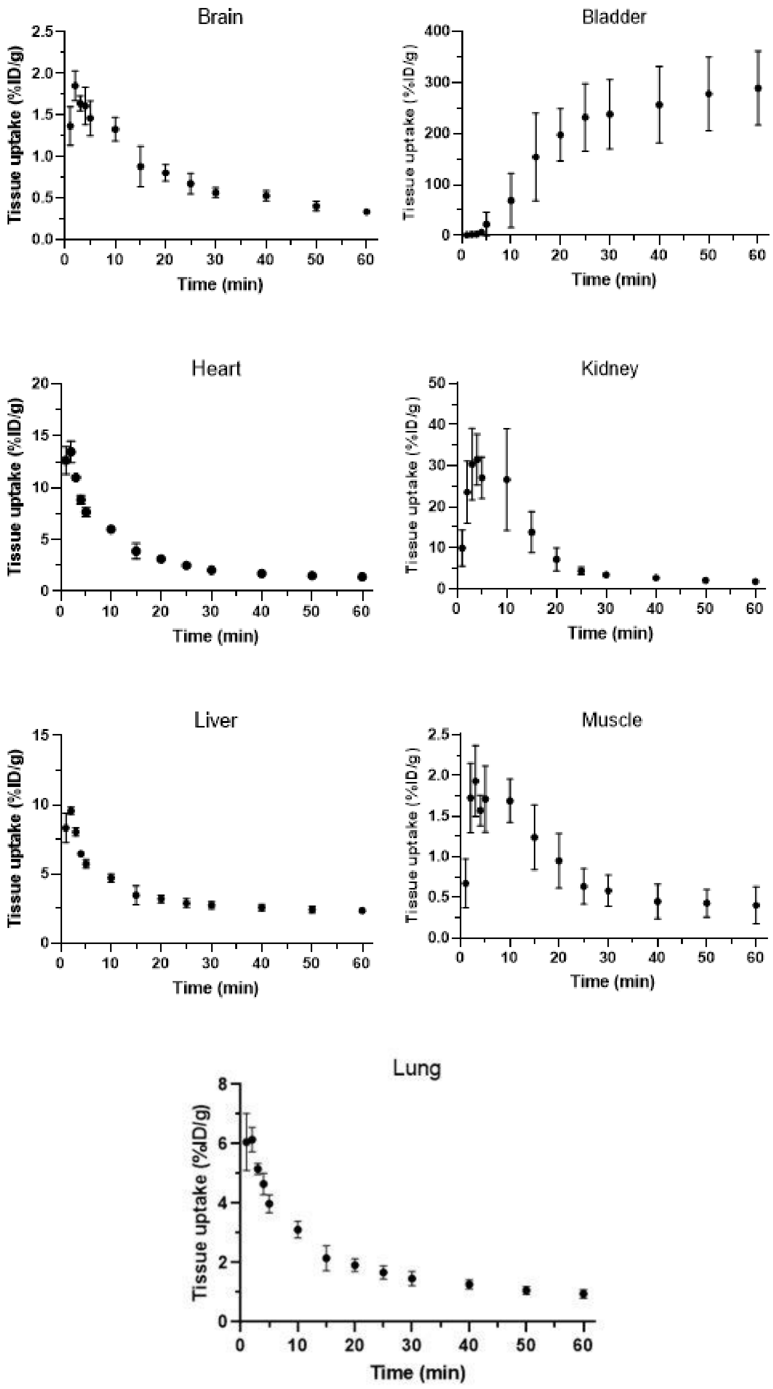

Figure S8. *In vivo* PET quantification of ROI: [^{68}Ga]Ga-SKN in healthy adult mice in different organs shows the pharmacokinetics of the radiotracer over time. This is cleared rapidly from the blood and is renally cleared to the bladder. Data are the means of four replicates ± standard deviation.

Urine sample analysis by RP radio-HPLC and radio-iTLC collected 60 min post-injection

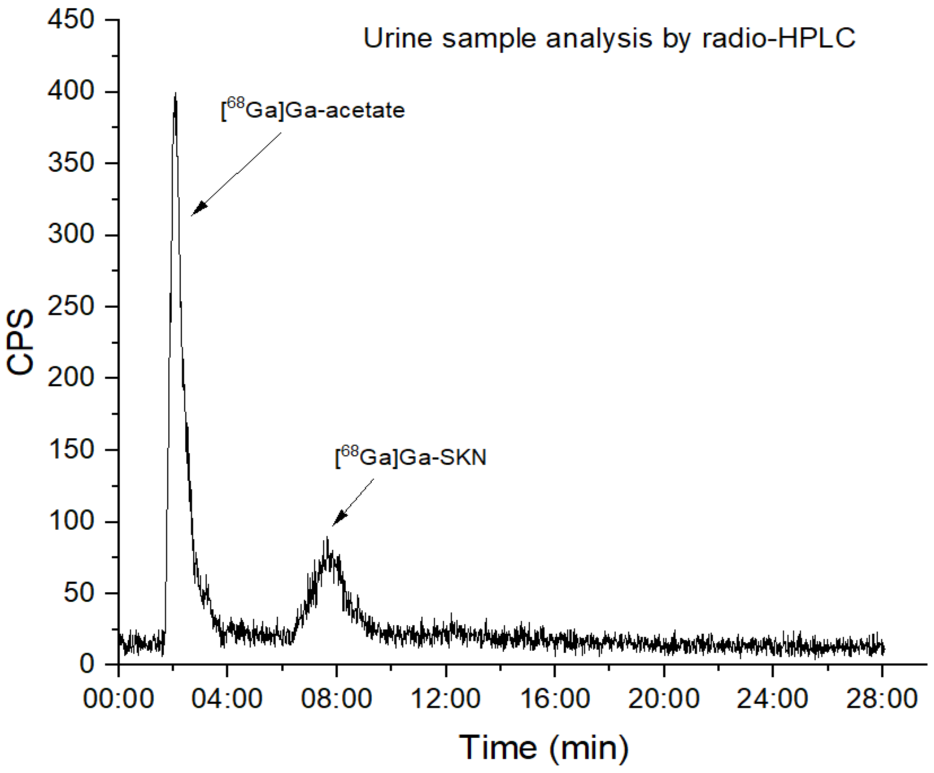

iTLC-SG of the same urine sample

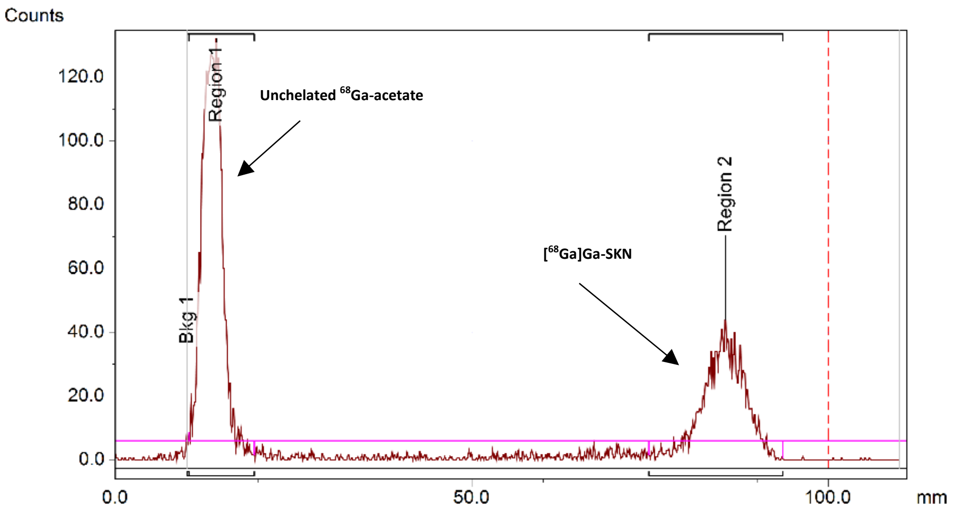

Figure S9. Stability of [^{68}Ga]Ga-SKN in urine collected after 60 min post-injection (p.i.) from a healthy mouse analyzed by RP radio-HPLC and radio iTLC (2.3 hr post-injection).

References

(1) Afnan M. F. Darwesh. Gallium-Essential Applications of Gallium-67 and Gallium-68. , King's College London , London , 2021.

(2) Akter, A.; Lyons, O.; Mehra, V.; Isenman, H.; Abbate, V. Radiometal Chelators for Infection Diagnostics. *Frontiers in Nuclear Medicine* **2023**, *2*. https://doi.org/10.3389/fnume.2022.1058388.

(3) Petrik, M.; Franssen, G. M.; Haas, H.; Laverman, P.; Hörtnagl, C.; Schrettl, M.; Helbok, A.; Lass-Flörl, C.; Decristoforo, C. Preclinical Evaluation of Two 68Ga-Siderophores as Potential Radiopharmaceuticals for Aspergillus Fumigatus Infection Imaging. *Eur J Nucl Med Mol Imaging* **2012**, *39* (7). https://doi.org/10.1007/s00259-012-2110-3.

(4) Abdul-Tehrani, H.; Hudson, A. J.; Chang, Y. S.; Timms, A. R.; Hawkins, C.; Williams, J. M.; Harrison, P. M.; Guest, J. R.; Andrews, S. C. Ferritin Mutants of Escherichia Coli Are Iron Deficient and Growth Impaired, and Fur Mutants Are Iron Deficient. *J Bacteriol* **1999**, *181* (5). https://doi.org/10.1128/jb.181.5.1415-1428.1999.

(5) MEYER, J. M.; ABDALLAH, M. A. The Fluorescent Pigment of Pseudomonas Fluorescens: Biosynthesis, Purification and Physicochemical Properties. *J Gen Microbiol* **1978**, *107* (2), 319–328. https://doi.org/10.1099/00221287-107-2-319.

(6) Petrik, M.; Umlaufova, E.; Raclavsky, V.; Palyzova, A.; Havlicek, V.; Haas, H.; Novy, Z.; Dolezal, D.; Hajduch, M.; Decristoforo, C. Imaging of Pseudomonas Aeruginosa Infection with Ga-68 Labelled Pyoverdine for Positron Emission Tomography. *Sci Rep* **2018**, *8* (1). https://doi.org/10.1038/s41598-018-33895-w.

(7) Beasley, F. C.; Vinés, E. D.; Grigg, J. C.; Zheng, Q.; Liu, S.; Lajoie, G. A.; Murphy, M. E. P.; Heinrichs, D. E. Characterization of Staphyloferrin A Biosynthetic and Transport Mutants in *Staphylococcus Aureus*. *Mol Microbiol* **2009**, *72* (4), 947–963. https://doi.org/10.1111/j.1365-2958.2009.06698.x.

(8) Hu, C. J.; Bai, C.; Zheng, X. De; Wang, Y. M.; Wang, Y. Characterization and Functional Analysis of the Siderophore-Iron Transporter CaArn1p in Candida Albicans. *Journal of Biological Chemistry* **2002**, *277* (34). https://doi.org/10.1074/jbc.M204545200.

(9) Chuljerm, H.; Chen, Y.-L.; Srichairatanakool, S.; Hider, R. C.; Cilibrizzi, A. Synthesis and Iron Coordination Properties of Schizokinen and Its Imide Derivative. *Dalton Transactions* **2019**, *48* (46), 17395–17401. https://doi.org/10.1039/C9DT02731A.
